# Critical review: involvement of endoplasmic reticulum stress in the aetiology of Alzheimer's disease

**DOI:** 10.1098/rsob.180024

**Published:** 2018-04-25

**Authors:** Shoko Hashimoto, Takaomi C. Saido

**Affiliations:** Laboratory for Proteolytic Neuroscience, RIKEN Center for Brain Science, 2-1 Hirosawa, Wako, Saitama 351-0198, Japan

**Keywords:** Alzheimer's disease, endoplasmic reticulum stress, unfolded protein response, App-knockin, APP/PS1 transgenic

## Abstract

The endoplasmic reticulum (ER) stress response is regarded as an important process in the aetiology of Alzheimer's disease (AD). The accumulation of pathogenic misfolded proteins and the disruption of intracellular calcium (Ca^2+^) signalling are considered to be fundamental mechanisms that underlie the induction of ER stress, leading to neuronal cell death. Indeed, a number of studies have proposed molecular mechanisms linking ER stress to AD pathogenesis based on results from *in vitro* systems and AD mouse models. However, stress responsivity was largely different between each mouse model, even though all of these models display AD-related pathologies. While several reports have shown elevated ER stress responses in amyloid precursor protein (APP) and presenilin 1 (PS1) double-transgenic (Tg) AD mouse models, we and other groups, in contrast, observed no such ER stress response in APP-single-Tg or *App*-knockin mice. Therefore, it is debatable whether the ER stress observed in APP and PS1 double-Tg mice is due to AD pathology. From these findings, the roles of ER stress in AD pathogenesis needs to be carefully addressed in future studies. In this review, we summarize research detailing the relationship between ER stress and AD, and analyse the results in detail.

## 1. Alzheimer's disease

### 1.1. General information about Alzheimer's disease

In 2015, approximately 47 million people were estimated to have dementia, and it is speculated that this population will increase to greater than 130 million by 2050 [1]. Alzheimer's disease (AD) is the most common form of dementia, accounting for 60–70% of dementia cases, and is characterized by cognitive impairment and progressive neurodegeneration [2]. To protect the health of elderly people and to reduce the burden of caregiving, new and effective therapeutic strategies to treat AD need to be developed as a matter of urgency.

The neuropathological hallmarks of AD include extracellular deposits of amyloid-β (Aβ) as the major component of senile plaques and neurofibrillary tangles composed of hyperphosphorylated tau protein. According to the amyloid cascade hypothesis, the deposition of Aβ in the brain is a central event and a primary trigger of AD pathogenesis [3]. Aβ is generated from the cleavage of amyloid precursor protein (APP) through sequential proteolytic cleavages mediated by two aspartic proteinases, β-secretase (BACE1) and γ-secretase. γ-secretase is a membrane-associated complex consisting of the following four different proteins: presenilin-1/2 (PS1/2), nicastrin, Aph1 and Pen2 [4]. The catalytically active site of γ-secretase resides within PS1/2, while the other proteins provide molecular support or stabilization. Genetic mutations in the *App* and *presenilin1/2* (*PSEN1/2*) genes are linked to early-onset familial AD (FAD). These disease-related mutations increase Aβ levels or change the properties of Aβ to more toxic forms.

Aβ is degraded by enzymes including neprilysin [5–7], insulin-degrading enzyme [8–10], endothelin-converting enzyme 1/2 [11] and Kallikrein-related peptidase 7 [12,13]. Importantly, neprilysin, which our group has identified as a potent Aβ-degrading enzyme, declines in the human brain with ageing, an outcome which may contribute to the increased Aβ pathology [14,15]. In addition to degradation by enzymes, secreted Aβ is cleared from the brain through the cerebrospinal fluid and further transported to the vascular system. Low-density lipoprotein receptor-related protein 1, which is located in the membranes of endothelial cells, is involved in the uptake of Aβ from parenchymal to endothelial cells, while P-glycoprotein, ABCG2 and ABCC1 participate in the translocation of Aβ from endothelial cells to the blood [16–19]. As sporadic AD (SAD) patients do not exhibit genetic anomalies associated with the mechanism of Aβ production, an imbalance between Aβ production and degradation might be a trigger for Aβ accumulation.

Microtubule-binding protein tau (MAPT, tau) is another important contributor to the aetiology of AD. Under physiological conditions, tau is a soluble and unstructured protein that co-localizes and stabilizes microtubules in the axon [20]. In AD and tauopathy, an intracellular accumulation of hyperphosphorylated tau was observed in the neocortical and hippocampal areas [21]. Abnormal post-translational modifications, including hyperphosphorylation, acetylation, glycosylation and nitration, are considered to cause the dissociation of tau from microtubules and subsequent misfolding [22]. Further to this, the pathological spread of pathological tau is correlated with Braak staging in AD [23]. A number of reports have observed a cell-to-cell transfer of pathological tau protein in cultured cells and mouse models [24]. Such diffusion of pathological tau is considered to be a cause of neurodegeneration in tauopathy-related neurodegenerative diseases. The findings of studies on frontotemporal dementia and Parkinsonism linked to chromosome 17 (FTDP17) suggested a direct interaction between tau pathology and neurodegeneration. Several mouse models that possess FTDP17 mutations display brain atrophy accompanied by neuronal loss [21]. As tau pathology appearing after amyloid pathology is well correlated with neurodegeneration in AD, the mechanism by which amyloid pathology is linked to tau pathology is considered to be one of the most important issues to be addressed [25].

### 1.2. Calcium signalling in Alzheimer's disease

Accumulated evidence has shown that Aβ oligomers or fibrils lead to neurotoxicity. The molecular mechanisms by which Aβ oligomers elicit neurotoxicity include the binding of extracellular Aβ oligomers to cell surface receptors and subsequent disruption of signal transduction. Disruption of the Ca^2+^ permeability of cells via surface receptors is considered as one of the major mechanisms of neurotoxicity caused by Aβ oligomers [26]. Several studies have demonstrated interactions between Aβ and various Ca^2+^-conducting channels, including those linked to glutamate receptors (AMPA and NMDA receptors; AMPAR and NMDAR, respectively), nicotinic acetylcholine receptor (AChR)-linked channels, P-/Q-type voltage-gated Ca^2+^ channels, and intracellular inositol trisphosphate receptor- (IP3R), dopamine receptor- and serotonin receptor-linked channels [27–30]. In particular, because AMPAR, NMDAR and AChR play important roles in cognitive functions in the hippocampus and neocortex, disruption of these receptor-mediated Ca^2+^-signalling pathways by Aβ could be responsible for AD symptoms. Indeed, these receptors are targets of currently licensed therapeutic agents [31].

NMDAR is associated with synaptic plasticity that regulates long-term potentiation (LTP) and long-term depression (LTD). A number of studies have demonstrated that LTP is impaired and LTD enhanced in AD or by exposure to Aβ [27,32–35]. The Aβ-induced perturbation of NMDAR elevates cytoplasmic Ca^2+^ levels and disrupts downstream pathways involved in synaptic function and neuronal cell death. For example, the abnormal activation of calcineurin/protein phosphatase 2B (PP2B) by Aβ oligomers via NMDAR induces several signal transduction processes including tau phosphorylation by GSK3β, internalization of AMPAR, synaptic collapse due to hyperactivation of neuronal/astroglial nuclear factor of activated T cells and depolarization of F-actin [36–40]. In addition, several groups have demonstrated that Aβ oligomers can cause neuronal cell death by promoting tau-Fyn kinase–PSD95 complex formation in post-synaptic sites [41,42]. Tau knockout (KO) mice have been reported to exhibit decreased levels of Fyn in neuronal dendrites and a reduced susceptibility to excitotoxicity induced by Aβ [41]. Moreover, the upregulation of reactive oxygen/nitrogen species (ROS/RNS) and mitochondrial dysfunction were reported to be induced by Aβ oligomers via NMDAR [43]. An abnormal activation of neuronal nitric oxide synthase (nNOS) together with mitochondrial Ca^2+^ overload generates an excess of ROS and RNS [44]. This leads to aberrant s-nitrosylation, sulfonation and accumulation of peroxides, resulting in protein dysfunction [44–46]. Mitochondrial dysfunction also induces caspase activation and cell death [47].

As we shall see below, the ER has numerous functions, one of which is as an intracellular Ca^2+^ reservoir. In neuronal cells, the ER extends from the soma towards the axons, dendrites and dendritic spines [48]. The ER plays a role in maintaining the cytosolic Ca^2+^ concentration by removing/releasing Ca^2+^ from/to the cytosol via Ca^2+^ channels located on the ER membrane. Sarcoplasmic/endoplasmic reticulum Ca^2+^ ATPase (SERCA) actively mediates the uptake of Ca^2+^ into the ER, whereas IP3Rs and ryanodine receptors (RyRs) mediate Ca^2+^ release from the ER [49–52]. Stromal interaction molecule (STIM) proteins (STIM1 and 2) act as ER Ca^2+^ sensors, promoting Ca^2+^ influx from the extracellular space via Ca^2+^ channels in the plasma membrane when low Ca^2+^ levels in the ER are sensed [53–55]. Several reports have demonstrated a dysregulation of ER Ca^2+^ influx and sensing in post-mortem AD samples and AD models. In addition, cross-talk between an Aβ-induced aberration of Ca^2+^ influx via cell surface receptors and ER Ca^2+^ homeostasis has also been reported. For example, increased RyR2 levels in hippocampal regions compared with healthy controls are seen in early-stage AD and mild cognitively impaired patients [56,57]. AD mouse models, including PS1-M146 V-KI (knockin) and TgCRND8 (APP695 including Swedish and Indiana mutants), also show increased RyR levels [58]. Moreover, other groups investigating pathologies in APP/PS1 X RyR3^−/−^ mice noted that deletion of RyR3 in young APP/PS1 mice resulted in enhanced AD pathology, while older mice exhibited reduced AD pathology. These results suggest that increased RyRs at an early stage are protective, whereas decreased RyR levels at a later stage worsen the AD pathology. As for ER Ca^2+^ sensing, Garcia-Alvarez *et al.* [59] demonstrated that specific STIM1 and STIM2 double-KO in the forebrains of mice impaired spatial memory, suggesting that STIM proteins are key regulators of protein kinase A signalling and synaptic plasticity in neural circuits encoding spatial memory [59]. Bezprozvanny's group showed decreased STIM2 expression in hippocampal neurons of PS1-M146V-KI and *App*-KI (*App*-knockin; below-mentioned) mice and in post-mortem samples from AD patients [60,61]. They further demonstrated that downregulation of STIM2 and store-operated calcium entry (nSOC), a cell surface Ca^2+^ channel controlled by STIM2, led to the loss of mushroom spines in hippocampal neurons [61]. Mushroom spines have a larger head and are involved in long-term memory storage [62]. They also proposed that extracellular Aβ over-activated the cell surface mGluR5 receptor, leading to elevated Ca^2+^ in the ER and downregulation of STIM2 and nSOC [60,61]. These findings suggest that ER Ca^2+^ homeostasis may be affected by extracellular Aβ via cell surface receptors. As the perturbation of ER Ca^2+^ homeostasis induces ER stress, ER stress could therefore be considered a plausible mechanism by which Aβ oligomers cause cell injury.

## 2. Endoplasmic reticulum stress in Alzheimer's disease

### 2.1. Unfolded protein response

**Figure 1.**
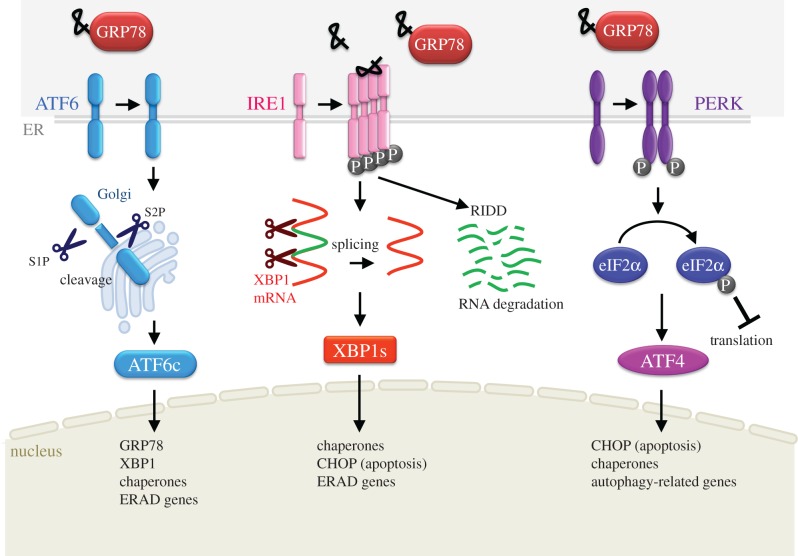
Summary of unfolded protein response. Canonical pathway of ER stress response. ATF-6, IRE1 and PERK act as sensor proteins of ER stress.

ER stress is regarded as an important aspect of the aetiology of AD. The accumulation of misfolded proteins and perturbation of intracellular Ca^2+^ homeostasis are thought to underlie the induction of ER stress, resulting in neuronal dysfunction and cell death. Under stress conditions, cells evade serious damage by activating adaptive response pathways known as the unfolded protein response (UPR). UPR activates three key pathways via three ER membrane-associated proteins: pancreatic ER kinase (PERK), activating transcription factor-6 (ATF-6) and inositol-requiring enzyme-1 (IRE1) (figure 1). Glucose-related protein 78 (GRP78/BiP) is a master sensor to initiate UPR via the three key pathways [63]. Under normal conditions, GRP78 is localized to the ER lumen, and PERK, ATF-6 and IRE1 remain in an inactive state due to GRP78 binding. Upon ER stress, misfolded proteins inhibit interaction between GRP78 and sensor proteins, thereby initiating UPR signalling.

PERK phosphorylates eukaryotic translation initiation factor 2α subunit (eIF2α) to inhibit its translation initiation activity [64,65]. Inhibition of eIF2α by phosphorylation mitigates ER burden by decreasing the amount of newly synthesized proteins. Further to this, PERK activates ATF-4, which turns on gene expression for the synthesis of ER chaperones and autophagy-related proteins. Collapse of membrane lipid homeostasis also induces ER stress. PERK is revealed to be activated by an imbalance of membrane lipid saturation [66].

ATF-6 migrates from the ER to the Golgi apparatus during ER stress and is processed by S1/S2P protease in the Golgi [67,68]. The cleaved fragment (ATF-6c), which contains a basic leucine zipper (bZIP) transcriptional activator, translocates to the cell nucleus to upregulate the synthesis of ER molecular chaperones such as GRP78 and GRP94, and protein-folding enzymes such as protein disulfide isomerases (PDIs). In addition to ATF-6, five species of homologous proteins, which belong to OASIS family, have been identified [69]. All OASIS family proteins are cleaved in the Golgi and their N-terminal fragment acts as transcription factors [69]. Almost all of them are specifically expressed in particular cell types and play roles in cellular function and differentiation [69,70].

IRE1 is dimerized via its luminal domain in response to ER stress [71]. Dimerization stimulates IRE1 autophosphorylation, with IRE1 gaining endoribonuclease activity that cleaves off an intron in the pre-mRNA of X-box-binding protein-1 (XBP1) [72]. XBP1 is a transcription factor that stimulates the expression of genes related to protein folding, autophagy and apoptosis (such as C/EBP homologous protein (CHOP)) [73,74]. IRE1 also degrades ER-derived mRNAs and inhibits translation initiation of nascent proteins. This process is called regulated IRE1-dependent decay [75]. The recent study revealed that IRE1 RNase activity also enhances decay of select microRNAs involved in repression of caspase-2 mRNA translation. This elevates caspase-2 protein levels and initiates mitochondrial apoptotic pathway [76]. In addition, IRE1 activates the pro-apoptotic pathway through complex formation with TNF receptor-associated factor 2 and apoptosis signal regulating kinase1 [77,78]. This complex enhances apoptosis by activating several downstream signalling pathways, including nuclear factor kappa B, c-Jun N-terminal kinase, caspase-12 and p38 mitogen-activated protein kinase (p38MAPK)-mediated CHOP activation [79–81]. A number of studies have detected upregulation of UPR signalling in *in vivo* and *in vitro* models of neurodegenerative disease, including AD, Parkinson's disease, amyotrophic lateral sclerosis, prion disease and polyglutamine diseases [82].

### 2.2. Relationship between endoplasmic reticulum stress and Alzheimer's disease

One of the pioneering works of ER stress–AD research demonstrated that PS1 mutations affect UPR in response to ER stress. We provide details of links between PS1 and ER stress in §3.2 below. Other studies have suggested that exposure of cells to Aβ activated caspase-12, which is a mouse homologue of human caspase-4 and functions as an ER-specific caspase, resulting in the induction of neuronal cell death [83–85]. Moreover, it was demonstrated that caspase-12-KO mice were resistant to ER stress and cell death caused by Aβ protein [86]. From these findings, ER stress was considered to be involved in neuronal cell death in AD. Following on from this, numerous studies using *in vitro* systems, AD animal models and human AD samples have examined the relationship between AD aetiology and UPR signalling.

**Figure 2.**
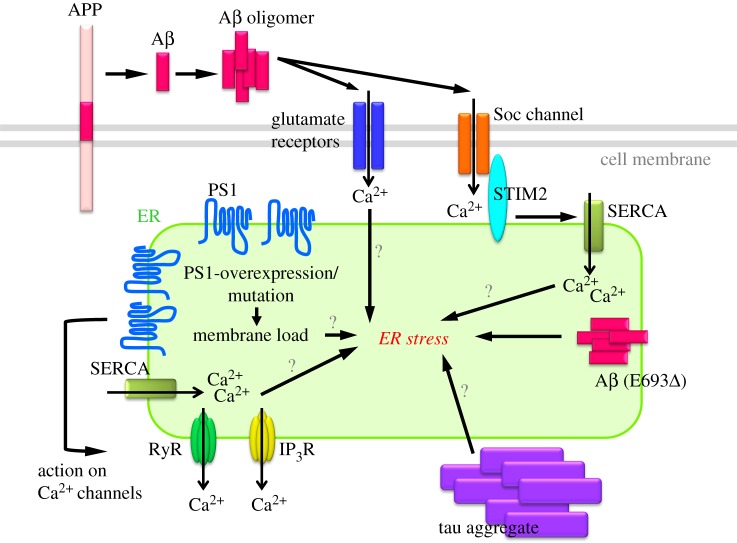
Summary of ER stress in AD models. The events considered as the causes of ER stress induction in AD models are summarized.

A number of reports have indicated that Aβ oligomers or fibrils trigger ER stress in *in vitro* experimental systems based on primary cultures of neuronal cells, cell lines and brain slices (figure 2) [87–93]. Further investigations have proposed mechanisms establishing a connection between extracellular Aβ and intracellular ER. The most likely mediator between Aβ and ER stress is Ca^2+^, with the binding of Aβ to glutaminergic receptors likely to induce ER stress-dependent cell death by disrupting cytosolic Ca^2+^ homeostasis. Indeed, in mature hippocampal cultures, treatment with Aβ oligomers elevates ER stress downstream from NMDARs [94]. A further report indicated that Aβ-induced early Ca^2+^ release through RyR and IP_3_R perturbed Ca^2+^ homeostasis and increased ROS production, leading to caspase-3-related cell death [95]. Alberdi *et al.* [88] showed that Aβ oligomers also induced astrocytic ER stress by disrupting Ca^2+^ signalling and astrogliosis. Casas-Tinto *et al.* [96] used spliced XBP1-overexpressed *Drosophila* and cultured cells to demonstrate that XBP1 prevented Aβ toxicity by inhibiting cytosolic Ca^2+^ accumulation. Moreover, a compound, cyanidin, has been identified that inhibits Aβ-induced cytotoxicity by attenuating Ca^2+^ imbalance in the ER [97]. Mitochondrial dysfunction and ROS production have also been identified as mediators of Aβ-induced ER stress and cytotoxicity. The cytochrome *c* oxidase-induced inhibition of mitochondrial damage in AD patients reduces cellular resistance to Aβ-induced ER stress [98]. Barbero-Camps *et al.* [99] indicated that Aβ-mediated ER stress and increased mitochondrial cholesterol trafficking contributed to the progression of pathology observed in aged APP/PS1 mice [99].

In addition to Ca^2+^ imbalance, a correlation between ER stress and APP mutation has been reported. Several kinds of FAD-linked APP mutations inhibit Aβ secretion to the extracellular space. The E693Δ (Osaka) APP mutation, which has been suggested to cause dementia, is associated with markedly altered Aβ trafficking and causes Aβ accumulation in the ER. Studies using induced pluripotent stem cells from an E693Δ APP carrier suggested that the mutation causes ER stress-induced cytotoxicity via enhancement of its intracellular oligomerization (figure 2) [87,100]. However, as only a small proportion of FAD patients have APP mutations that cause abnormal localization, the link between this mutation and ER stress does not apply to most AD patients.

Tau pathology has also been postulated to induce ER stress (figure 2) [101–103]. A study using tau transgenic rTg4510 mice reported that tau's interaction with the ER membrane impaired ER-associated degradation (ERAD) and activation of the UPR [103]. Conversely, several reports have indicated that ER stress exacerbates pathology as a consequence of the delayed degradation of tau protein due to decreased binding between tau and the carboxyl terminus of Hsc70-interacting protein [104], thereby facilitating tau hyperphosphorylation [105–108]. From these findings, ER stress and tau pathology are considered to form a vicious cycle that gives rise to neuronal cell death.

### 2.3. Endoplasmic reticulum stress in Alzheimer's disease mouse models and human Alzheimer's disease samples

A number of studies have shown an upregulation of ER stress markers in AD models. Table 1 summarize UPR responses in representative AD and tauopathy mouse models (table 1). In the APP/PS1 mouse, which overexpresses APP (Swedish) and PS1 (ΔE9), higher levels of GRP78, p-PERK, p-eIF2α, CHOP and ATF-4 are seen [99,111]. Moreover, Ma *et al.* [111] showed that the genetic deletion of PERK, which inhibits eIF2α phosphorylation, prevented deficits in protein synthesis, synaptic plasticity and spatial memory in APP/PS1 mice. Another report, however, showed no effects of eIF2*α*-S51A knockin, which expresses non-phosphorylatable eIF2α, on 5XFAD mouse behaviour except for locomotor hyperactivity [120]. In the 5XFAD model, which overexpresses APP (Swedish/Florida/London) and PS1 (M146 L/L286 V), higher levels of p-eIF2α and spliced XBP1 mRNA are displayed [112,115]. Using the 5XFAD mouse model, O'Connor *et al*. [115] proposed that eIF2α phosphorylation increases BACE1 levels and that this causes Aβ overproduction, which could be a mechanism underlying SAD. Further to this, Reinhardt *et al*. [112] demonstrated that increased XBP1 splicing in young 5XFAD mice enhances ADAM10 (α-secretase) gene expression, but that an age-dependent loss of spliced XBP1 and a decline in ADAM10 induce Aβ overproduction. By contrast, Lee *et al*. [110] observed no UPR signals in Tg2576 mice, which overexpress APP (Swedish) but not PS1. Given that all of the above mouse models display progressive Aβ pathology, why do results differ so markedly between them? Are Aβ and related pathologies the real cause of ER stress?

**Table 1.** Summary of ER stress responses in representative AD mouse models. Expression levels of UPR-related genes in AD mouse models are summarized. #M signifies #-month-old and *M*/*F* refers to male/female.

gene modification	line	age (months)	ER stress markers	up or down	brain region	references
*App*	*App*NL-G-F	6	GRP78	→	hippocampus/cortex	Hashimoto *et al*. [109]
			p-eIF2α	→		
			PDI	→		
			CHOP	→		
			XBP1 splicing	→	cortex	
		14	GRP78	→	cortex	
			p-eIF2α	→		
			PDI	→		
			CHOP	→		
			XBP1 splicing	→		
	Tg2576	17	GRP78	→	—	Lee *et al*. [110]
			PDI	→		
			CHOP	→		
		6	GRP78	→	hippocampus/cortex	Hashimoto *et al*. [109]
			p-eIF2α	→		
			PDI	→		
			CHOP	→		
			XBP1 splicing	→		
	APP23	6	GRP78	→	hippocampus/cortex	Hashimoto *et al*. [109]
			p-eIF2α	→		
			PDI	→		
			CHOP	→		
			XBP1 splicing	→		
*App*/*PSEN1*	APP/PS1 [APP(Swe)-Tg,PS(ΔE9)-Tg]	10–12	p-eIF2α	↑	hippocampus	Ma *et al*. [111]
			ATF-4	↑		
		4, 7, 10	GRP78	↑(age-dependent)	—	Barbero-Camps *et al*. [99]
			CHOP	↑(age-dependent)		
			p-PERK	↑(age-dependent)		
			p-eIF2α	↑(age-dependent)		
		2, 6, 9	spliced Xbp1	2 months→, 6 months↑, 9 months↓	—	Reinhardt *et al*. [112]
			Ire1α mRNA	2 months→, 6 months↑, 9 months↓		
		6	GRP78	↑	hippocampus	Cui *et al.* [113]
			CHOP	↑		
		6, 15	GRP78	→	hippocampus/cortex	Hashimoto *et al*. [109]
			p-eIF2α	↑		
			PDI	→		
			CHOP	→		
			XBP1 splicing	→	cortex	
	APP/PS1-KI [APP(Swe)-Tg, PS(M233T/L235T)-KI]	—	p-eIF2α	↑	cortex	Mouton-Liger *et al*. [114]
	5XFAD	6	p-eIF2α	↑	hemibrain	O'Connor *et al*. [115]
		1, 2, 9	spliced Xbp1	1 month↑, 2 months→, 9 months↓	—	Reinhardt *et al*. [112]
			p-Ire1	1 month↑, 9 months↓		
*Mapt*	P301S-Tg (C57BL/6 background)	3, 6, 9, 12, 15 (cortex), 12 (hippocampus)	GRP78	→	hippocampus/cortex	Hashimoto *et al*. [109]
			p-eIF2α	→		
			PDI	→		
			CHOP	→		
			XBP1 splicing	→		
	P301S-Tg (PS19)	4	p-PERK	→	hippocampus	Kim *et al*. [108]
	rTg4510	9	GRP78	↑	—	Abisambra *et al*. [103]
			p-PERK	↑		
		6	p-eIF2α	↑		Radford *et al*. [116]
			ATF-4	↑		
		4	p-PERK	↑	hippocampus	Kim *et al*. [108]
			p-eIF2α	↑		
			CHOP	↑		
	rTg21221	4, 8	CHOP	↑	hippocampus	Kim *et al*. [108]
*App/PSEN1/Mapt*	3XTg	2	GRP78	↑	—	Soejima *et al*. [117]
		3, 12	GRP78	3 months: male→, female↑; 12 months: male↑, female→	cortex	Mota *et al*. [118]
			XBP1 protein	3 months: male→, female↑; 12 months, male/female→		
			CHOP	3 months: male/female→		
		21	GRP78	↑	hippocampus	Hashimoto *et al*. [109]
			p-eIF2α	→		
			PDI	→		
			CHOP	↑		
	TauPS2APP (pR5)	18–24	p-PERK	↑	neurons with an early stage of tau hyperphosphorylation	Köhler *et al*. [119]

As for an amyloid/tauopathy mixed mouse model, increased GRP78, XBP1 and CHOP have been detected in the 3XTg mouse, which overexpresses APP (Swedish) and tau (P301 L) transgenes on a PS1 (M146V)-KI background [117,118]. However, expression levels of UPR-related genes were different between male and female animals [118]. A recent study showed that XBP1 restored hippocampal synaptic plasticity and memory by controlling the Kalirin-7 pathway in 3XTg mice [121]. The TauPS2APP (pR5) mouse, which overexpresses APP (Swedish), PS2 (N141I) and tau (P301 L), displays higher levels of p-PERK and ATF-4 in neurons with AT100-positive phosphorylated tau [119]. Moreover, concerning single-Tau-Tg mouse models, it has been shown that rTg4510 mice, which overexpress tau (P301 L), exhibit increased levels of p-PERK, p-eIF2 and ATF-4 [103,116]. Abisambra *et al.* [103] also proposed that increased levels of ubiquitinated protein were accompanied by PERK phosphorylation, and Radford *et al.* [116] showed that PERK inhibitor prevents tau-mediated neurodegeneration in rTg4510 mice. Expression levels of UPR-related genes in three tauopathy mouse models, such as rTg4510, rTg21221 (WT-human tau-Tg) and PS19 (P301S-Tau-Tg), were provided in another report [108]. Among them, rTg4510 showed upregulation of p-PERK, p-eIF2α and CHOP; rTg21221 had upregulated levels of CHOP, while PS19 had no alteration in p-PERK or CHOP levels. Similarly, Spatara & Robinson [122], who showed no activation of UPR in PS19 mice, doubted a direct mechanistic link between tau aggregation and UPR. Taken together, because discrepancies exist between the different mouse models, the link between tau pathology and ER stress is also somewhat unclear.

Many groups have reported upregulation of the ER stress response in post-mortem human AD brains [101,102,114,115,123–125]. For instance, upregulation of p-eIF2a, PERK, CHOP and PDI in AD samples was detected through western blot or immunohistochemical analyses by several groups. Hoozemans *et al.* [124] observed upregulation of GRP78 in the hippocampus and temporal cortex. They also showed that the number of p-PERK-positive neurons increases in line with the Braak stage for neurofibrillary changes [101]. Further to this, formations of inclusions with or without amyloid plaques or tau aggregates were detected in association with GRP78, PDI and CD3-delta, which are ERAD substrates [103,117,126]. By contrast, Katayama *et al*. [127] observed a significant decrease of GRP78, while Reinhardt *et al*. [112] showed a decrease of spliced XBP1 in the brains of AD patients.

Taken together, the degree of UPR in human AD samples, as evidenced by ER stress markers, is also inconsistent. Care should be exercised in the analysis of post-mortem samples as the post-mortem degradation of mRNA and protein might be different between control and AD patients. For example, neurons in post-mortem AD brains have undergone prior degeneration, which would be accompanied by damage to lysosomes and mitochondria, before sampling. Moreover, Ca^2+^ concentrations and Ca^2+^-related responses might also be altered by post-mortem conditions. To this end, we have shown a non-physiological activation of the Ca^2+^-dependent protease calpain in post-mortem mouse brains [128]. It is thus difficult to discuss ER stress in post-mortem human samples. The results obtained using human samples have been poorly reproduced. We assume that the poor reproducibility is due to variation of samples' backgrounds (e.g. stage of AD progression, medical history, brain region, age and sex). Differences of these backgrounds might influence the cellular ability of stress responses. We cannot be convinced by the upregulation of ER stress in human AD brain without validations using a number of biopsied human samples.

## 3. Is endoplasmic reticulum stress in Alzheimer's disease models real?

### 3.1. Artificial overexpression of amyloid precursor protein and presenilin 1

As the clinical features and pathological processes of FAD and SAD are highly similar, most research progress has been made based on studies using animal models possessing FAD-linked mutation(s). In the basic and clinical studies of AD, APP- and/or PS1-overexpressing (transgenic, Tg) or mutation-KI mice have been used widely as AD mouse models [129]. While APP (and PS1) overproduction increases amyloid deposits, this approach may generate side effects via unforeseen mechanisms. One reason for this is that the processes that give rise to high levels of Aβ in conventional mouse models differ markedly from physiological processes in AD patients. APP overexpression produces fragments other than Aβ, such as soluble APP (sAPP), C-terminus fragment of APP and APP intracellular domain (AICD), at unphysiologically high levels. Overproduction of these fragments could induce artificial effects beyond the true AD pathogenesis. For example, Li *et al.* [130] demonstrated that sAPP fragments modulated transthyretin and Klotho gene expression levels. Although the mechanisms of AICD function in gene regulation remain controversial, several reports have demonstrated the transcriptional activity of AICD, which can form transcriptionally active complexes with the nuclear adaptor Fe65 and the histone acetyltransferase Tip60 [131]. The established genes regulated epigenetically by AICD include neprilysin, transthyretin and stathmin-1 [132–134]. Disruption of these functions of APP fragments by APP overexpression could lead to artificial phenomena and phenotypes. A second effect is the possibility of induction of artificial ER stress by overexpression of membrane protein(s). APP and PS1 are one- and nine membrane-spanning proteins, respectively. We therefore inferred that overexpressed membrane proteins can become wedged in a misfolded structure in ER membranes, thereby inducing artificial ER stress.

To circumvent these drawbacks of the overexpression paradigm, we recently generated novel AD mouse models based on a knockin strategy [135]. The *App*-KI mouse expresses APP which includes the humanized Aβ sequence with familial AD mutations at endogenous levels. We produced two lines of the *App*-KI mouse model. The first (*App^NL−F^*) is knocked in with two mutations (Swedish and Iberian), while the second (*App^NL-G-F^*), which includes a further mutation (Arctic), shows an even faster onset of pathologies. These mice exhibit not only amyloid pathology but also neuro-inflammation and impaired memory. Interestingly, *App-*KI mice failed to reproduce several observations made with APP-overexpressing mouse models. We previously observed the early lethality of *Calpastatin*-KO X APP23 mice, which contradicted the chronic nature of AD. *Calpastatin*-KO X *App*-KI mice, however, did not show premature death [135,136]. Moreover, no calpain-dependent conversion of p35 to p25, which upregulates CDK5 activity, was observed in *App*-KI mice [128]. Although it is generally understood that calpain activation plays a pivotal role in the pathogenesis of AD due to its contribution to caspase-dependent neuronal cell death and CDK5-mediated tau phosphorylation, our findings indicate that the functions of calpain may have been overestimated.

### 3.2. Impact of presenilin 1 gene modifications on the manifestation of endoplasmic reticulum stress

As described above, PS1 contains nine transmembrane-spanning domains and is enriched in ER membranes associated with mitochondria [137]. As ER–mitochondria contact sites are active locations for Ca^2+^ transport and Ca^2+^ signalling, an elevated possibility exists that overexpression or genetic modification of PS1 will affect Ca^2+^ homeostasis and result in artificial ER stress (figure 2). Indeed, a number of studies have reported that PS1 functions in the regulation of ER Ca^2+^ transport and signalling [138,139]. PS1 regulates not only the function of SERCA but also ER-associated Ca^2+^ channels such as IP3R and RyR [140–144]. Moreover, FAD-linked mutations of PS1 alter its function in Ca^2+^ transfer [138,139]. Alteration of the ER and cytosolic Ca^2+^ concentration could induce ER stress, as seen in cells treated with the SERCA inhibitor thapsigargin, which is widely used as a potent ER stress inducer [145]. In addition, PS1 mutations alter expression levels or activities of STIM1/2, which are ER Ca^2+^ sensor proteins [61,146–148]. Therefore, ER stress could be enhanced simply by the overexpression or genetic modification of PS1 even if there are no AD-related pathologies (such as amyloid and tau pathologies) present.

Indeed, several studies have demonstrated altered ER stress responses as a consequence of PS1 gene modifications. Niwa *et al.* [149] reported suppression of IRE1 signalling under ER stress conditions in PS1 KO fibroblasts and further indicated that PS1 controlled IRE1 proteolysis in mammalian cells. By contrast, one study showed upregulation of GRP78, PDI, CHOP and ATF-6 by knockdown of PS1 in Hep3B cells [150]. As for FAD mutations, overexpression of PS1, including the ΔE9 mutation, enhances ER stress and caspase-4-dependent cell death [84]. A further report demonstrated that two kinds of FAD-linked PS1 mutants (L286 V and M146 V) expressed in PC12 cells and KI mice induced increased levels of p-eIF2α and CHOP [151]. By contrast, one group proposed that, in cell lines and primary cultured neurons of mutant PS1-KI mice, the expression of some PS1 mutants attenuated UPR [127,152,153]. The reports claimed that a downregulated ER stress response leads to the accumulation of unfolded proteins and cytotoxicity. From these findings, although the ER stress response differs depending on studies or samples, modification of PS1 itself appears to affect ER stress responses.

While some FAD patients exhibit mutations of the PS1 gene, almost all AD patients do not have such mutations. Therefore, ER stress due to PS1 modification should not be considered as a generalized phenomenon. Accordingly, even if the ER stress response is detected in AD mouse models with genetically modified PS1, we cannot assume that the responses are causally associated with the aetiology of AD.

## 4. *App*-KI and APP-single-Tg mice do not exhibit an endoplasmic reticulum stress response

**Figure 3.**
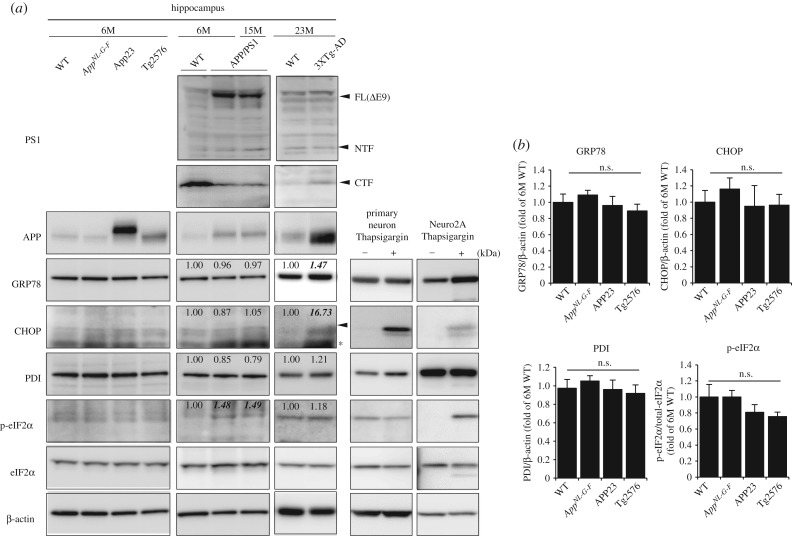
ER stress markers in *App^NL-G-F^* and APP-Tg mice. (*a*) Western blot analyses of ER stress markers in the hippocampi of six-month-old (M) WT, *App^NL-G-F^*, APP23 and Tg2576 mice. Expression in 6- and 15-month-old APP/PS1 and 23-month-old 3XTg-AD mice were also detected. Values shown in figures are the band intensity for each band which is normalized to β-actin values (for GRP78, CHOP and PDI) or total eIF2α (for p-eIF2α). As a positive control, ER stress markers in thapsigargin-treated primary cultured cortical neuronal cells or Neuro2a cells were confirmed. Arrowhead shows bands for CHOP, and asterisk shows non-specific bands. (*b*) Expression levels of ER stress markers were normalized to that of β-actin (for GRP78, CHOP and PDI) or total eIF2α level (for p-eIF2α), and reported as relative levels compared with expression in six-month-old WT mice. Data are shown as means ± s.e.m. (*n* = 3). Differences between groups were examined for statistical significance with one-way ANOVA. n.s.: no significant difference.

**Figure 4.**
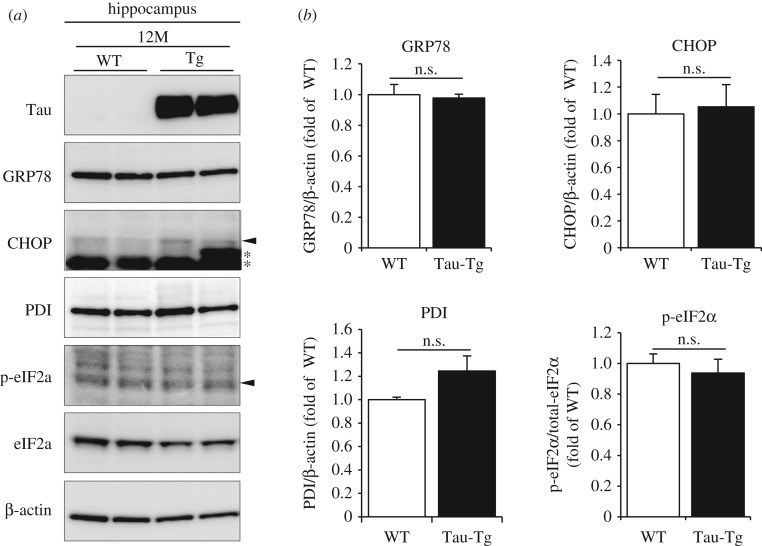
ER stress markers in P301S-Tau-Tg mice. (*a*) Western blot analyses of ER stress markers in the hippocampi of 12-month-old (12M) WT and P301S-Tau-Tg mice. Arrowhead shows bands for CHOP or p- eIF2α, and asterisks show non-specific bands. (*b*) Shown are mean levels ± s.e.m. of relative expression of ER stress markers (*n* = 3). Differences between groups were examined for statistical significance via two-way ANOVA. n.s.: no significant difference.

To determine whether the ER stress response is heightened because of Aβ pathology, we investigated ER stress in *App*-KI, APP-single-Tg and APP/PS1 double gene-modified AD mouse models [109] (figures 3 and 4). To verify the presence or absence of ER stress, we analysed several ER stress markers (GRP78, PDI, CHOP, p-eIF2α and spliced XBP1) in the models by western blotting analyses. First, to examine whether Aβ accumulation induces ER stress, we investigated levels of markers in the cortices of young and older *App^NL-G-F^* mice. No difference in any of the stress markers was observed between young/old wild-type (WT) and *App^NL-G-F^* mice, suggesting that increased Aβ deposition is not correlated with the ER stress response [109]. Moreover, we compared differences in the ER stress response between APP-Tg and *App*-KI mice. Unexpectedly, no alteration of ER stress markers was seen in APP-single-Tg (APP23 and Tg2576) mouse models [109] (figure 3 and table 1). As APP is a membrane-binding protein, we predicted that APP overexpression would induce chronic ER stress; however, there was no upregulation of the ER stress response in these animals. These results indicate that neither Aβ deposition nor APP overexpression enhances detectable ER stress. By contrast, APP and PS1 double gene-modified mice showed significant increases in the levels of some ER stress markers. The APP/PS1 mouse, which overexpresses APP (Swedish) and PS1 (ΔE9), exhibits higher levels of p-eIF2α. On the other hand, the 3XTg mouse exhibits elevated levels of GRP78, CHOP and p-eIF2α compared to age-matched WT controls. These results indicate that the genetic modification of PS1, or double modifications of APP and PS1, induced ER stress that is not related to the AD pathology. In our study, however, we did not detect other ER stress markers in APP/PS1 and 3XTg mice, whereas other studies have shown the upregulation of such markers in these mice. We consider that the partial reproducibility was perhaps due to decreased expression levels of APP and PS1 as a consequence of the number of passages.

In AD and tauopathy-related neurodegenerative diseases, tau pathology correlates well with neurodegeneration [154]. Under prolonged or severe ER stress conditions, cells stop protecting themselves from stress and activate cell death signals. Therefore, ER stress might be a mediator for tau-induced neuronal cell death. As described above, several studies have shown activation of the UPR in Tau-Tg mouse lines. Accordingly, we analysed ER stress markers in P301S-Tau-Tg mice on a C57BL/6 background; however, we observed no elevation of ER stress markers between 3- and 15-month-old animals [109] (data shown in figure 4 are for 12-month-old animals). These results suggest that tau pathology does not accompany ER stress, and that the ER stress response does not contribute to tau-induced neurodegeneration.

In the course of our studies, the principal conclusion we arrive at is that there is no relationship between AD aetiology and ER stress, and that the role of ER stress in the pathogenesis of AD needs to be carefully addressed in future studies.

## 5. Conclusion

A number of studies have indicated the contribution of ER stress to the pathogenesis of AD. From the point of view of Ca^2+^ homeostasis anomalies or protein misfolding in AD, ER stress could be regarded as a plausible mechanism leading to cell injury. However, discrepancies between studies cannot be ignored, and risks are associated with the use of overexpression paradigms for ER stress studies. In our research, we have raised serious concerns surrounding efforts to translate basic findings obtained using APP/PS1 gene-modified mice to clinical applications. We advocate that PS1-modified mice, in particular, are not appropriate for studies of ER stress and related events. Choosing appropriate models is thus essential if the molecular mechanisms underlying AD are to be elucidated [129].

## Data accessibility

This article has no additional data.
